# Qualitative study exploring the views and perceptions of parents/carers of young children with CF regarding the introduction of CFTR modulator therapy (The REVEAL study; PaRents pErspectiVEs of KAftrio in chiLdren aged 2–5)

**DOI:** 10.1136/bmjresp-2024-002522

**Published:** 2025-01-30

**Authors:** Sioned Haf Davies, Faye Wade, Heather Fogg, Adam Walsh, Kevin W Southern

**Affiliations:** 1 University of Liverpool School of Health Sciences, Liverpool, UK; 2 Alder Hey Children’s NHS Foundation Trust, Liverpool, UK; 3 Department of Women's and Children's Health, Institute of Life Course and Medical Sciences, Liverpool, UK

**Keywords:** Cystic Fibrosis, Paediatric Lung Disease

## Abstract

**Background:**

Cystic fibrosis (CF) is associated with a historically high treatment burden which causes anxiety and exhaustion for parents of children with CF, especially in the early years of a child’s life. Recently, a new medication, elexacaftor/tezacaftor/ivacaftor (ETI), has become available to some people with CF, which has had a significant impact on the quality of life of older children and adults. This medication will soon be available for children ages 2–5 in the UK. This study investigated parents’ perspectives before their children could start ETI.

**Method:**

10 parents of young children with CF participated in semistructured online focus groups. The data were analysed using thematic analysis to identify key themes.

**Results:**

Three reviewers identified four main themes: (1) The ‘roller coaster’ of parental emotions: Shock, hope, uncertainty and anticipation, (2) The dark side of the unknown, side effects and burden of decision making, (3) The value of simple pleasures in a life with CF; treatment burden, normality, future, family life and (4) Reforming clinical care in the new era of CF care; support, communication and the future.

**Conclusion:**

Parents experience a range of emotions from the day of diagnosis. While ETI brings hope and positivity, parents are concerned about the medication’s safety. Parents have clear hopes and wishes for their child’s future and reflect on the need for clinicians to consider reforming clinical care in the new era of CF for those eligible for new therapies.

**WHAT IS ALREADY KNOWN ON THIS TOPIC**

- Parental anxiety, chronic stress and physical exhaustion are often more pronounced during the early years of a child diagnosed with cystic fibrosis. Supporting parents is extremely important as medical needs change and the treatment burden increases. The benefit of a highly effective modulator therapy (elexacaftor-tezacaftor-ivacaftor (ETI)) and the hope it brings for adults and adolescents have been demonstrated.

**WHAT THIS STUDY ADDS**

- This is the first study to explore parents’ perceptions of young children under the age of 6 soon to start ETI. New therapies give hope to parents during a diagnosis period that is reported to be traumatic and overwhelming. Parents of young children with CF remain uncertain about the future and worry about the side effects of new therapies. Families hope to reduce the treatment burden in the future.

**HOW THIS STUDY MIGHT AFFECT RESEARCH, PRACTICE OR POLICY**

- During times of uncertainty, parents and carers wish for peer support and the support of others in similar situations. Ongoing support and clear, consistent communication by clinical teams are essential for parents and carers when introducing new therapies.

## Introduction

 Until recent years, the management of cystic fibrosis (CF) has focused on supportive therapies, such as physiotherapy treatments, antibiotic therapy and nutritional supplementation.^1^ Caregivers of children with CF, often parents, undertake daily treatments with their child and witness the ever-increasing treatment burden^2^ which can impact a caregiver’s quality of life.^3^ Previous studies of the lived experience of families of people with CF (pwCF) found that parental anxiety, chronic stress and physical exhaustion are often more pronounced during the early years of the child’s life.^4 5^ Families can experience significant psychosocial stressors, witnessing the changing medical needs of their child while coming to terms with the diagnosis.^4^ Parental support is essential during the child’s early years as better parental adjustment impacts the child’s quality of life, treatment adherence and engagement with the CF team.^6^

In the last few years, families and caregivers have seen the development of new therapies that target the basic defects within the CFTR protein that cause CF.^7^ This defect causes thick mucus and digestive fluids, leading to blockages, inflammation and an increasing risk of respiratory infections. Elexacaftor/tezacaftor/ivacaftor (ETI) is a modulator therapy which works by increasing the activity of the malfunctioning CFTR, correcting the underlying CF defect and improving clinical outcomes.^8^ Since the introduction of modulator therapy, parents of adolescents with CF found a sense of relief once they had confidence that the medication worked and benefited their child.^2^ Although the new medication led to a decreased treatment burden, some parents were reluctant to change their child’s treatment routine due to unknown long-term outcomes. A recent study by Landess *et al* found that the caregivers of children with CF between the ages of 7 and 16 years old described optimism for the future as a significant feeling in response to modulator therapy, while one caregiver described concerns for side effects as the primary drive not to commence ETI.^9^

In November 2023, a health technology assessment undertaken by the National Institute for Health and Care Excellence in the UK (NICE) produced draft guidance which concluded that, while clearly effective, ETI was not cost-effective at a current negotiated price. While not unexpected (given the high costs), this draft judgement was deeply unsettling for the CF community, particularly for parents of preschool children, as ETI had not yet been approved for this age group (NICE announced that pwCF already on ETI would remain on it). Subsequently, the UK government announced approval for ETI in this younger age group and the prescription became available in January 2024.

Caregivers conceptualise experiences differently from healthcare providers;^3^ therefore, it is vital that we capture families’ experiences and, as healthcare providers, deliver essential anticipatory guidance to support parents during the introduction of new therapies. There is a lack of literature capturing the lived experience of parents/caregivers during the introduction of ETI, a potentially life-changing intervention for their children. This paper will share some insight into the perspectives of families, to improve the introduction of future therapies with this young age group and help guide care teams in their support.

## Methods

### Participants and recruitment

53 families of children prospectively eligible for ETI (children aged 2–5) were approached by a regional Paediatric CF Service in Merseyside, Cheshire and North Wales between November 2023 and January 2024. Purposive sampling was used to include participants with unique experiences related to the research question and this was done to identify and recruit participants who can likely provide rich and diverse data. The inclusion criteria were a parent/caregiver of a child diagnosed with CF between the ages of 18 months and 6 years old, eligible for ETI while under the care of the Alder Hey Cystic Fibrosis Team and with access to an electronic device to take part in a virtual focus group. Clinicians approached all eligible participants during their regular appointments at their CF clinic and two recruitment emails were sent. If the participant expressed an interest, they were given a patient information sheet and asked to complete a paper consent form. Focus groups were conducted to investigate perceptions, expectations and realities of ETI.

### Data collection

15 parents expressed an interest in the study. As detailed in table 1, eight mothers and two fathers took part with children aged between 2 and 5. Three semistructured focus groups with a total of 10 parents were conducted between December 2023 and January 2024. Five parents were unable to attend the proposed dates and times. Families chose virtual focus groups as the most convenient method. The mean duration of the virtual focus groups was 60 min (ranging between 53 and 67 min). A topic guide was developed to facilitate the discussion. The focus groups focused on feelings and thoughts surrounding ETI, the expectations parents have for the medication and the realities (complete topic guide in online supplemental appendix 1). The focus groups were conducted and recorded via Microsoft Teams and then transcribed by a research team member (SHD).

**Table 1:** Participants’ demographic information

	Gender	Relationship to the child	Age of the child (at the time of the interview)	Focus group
P1	F	Parent	2	1
P2	M	Parent	2	1
P3	F	Parent	3	1
P4	F	Parent	4	2
P5	F	Parent	5	2
P6	F	Parent	4	2
P7	F	Parent	3	3
P8	F	Parent	2	3
P9	F	Parent	3	3
P10	M	Parent	3	3

### Data analysis

Following each focus group, the facilitator and one researcher (SHD) met to debrief. The debrief included discussing impressions of the group’s responses and highlighting the key issues. Two independent researchers (SHD, HF) reviewed transcripts for each focus group and compared results to review uniformity of interpretation. Key dimensions were recorded to create codes and led by developing overarching themes (see online supplemental material). Codes allowed the identification of themes and patterns among the participant’s experiences.^10^ Themes were discussed between two researchers and the significance of themes was determined. Any disagreements were to be discussed with a third author (AW); however, this was not required. Following each focus group, a summary was shared with participants to explore the accuracy and resonance of their experiences. No further comments from families were received.

### Reflexivity

In qualitative research, the researcher’s perspective can impact the knowledge construct and influence those participating.^11^ The data collector completed field notes during the focus group to reflect on interpersonal dynamics and highlight moments of analytical insights.^12^ A debrief was completed following each focus group between the data collector and a research team member to discuss the main themes and influence of prior assumptions on the data collection. The research team employed a facilitator who does not work within the CF clinical team to conduct the focus groups. This facilitator is a female physiotherapist (BSc) who underwent training with the research team. The facilitator (FW) had no prior contact with the participants and the participants were informed of why a member not from the clinical team had conducted the focus group to reduce prior assumptions. The researchers (SHD, HF) work within a CF multidisciplinary team (MDT) and discussed prior assumptions and how this might influence the data analysis process.

### Patient and public involvement

Families were involved in the study’s design, reflecting on burden and time commitments. All families who expressed interest preferred a virtual focus group over a face-to-face one. Families were also involved in the analysis of findings. Participants were encouraged to contribute different perspectives on comprehension and interpretation by sharing the initial themes.

## Results

Four overarching themes encapsulating the perceptions and experiences of parents and caregivers emerged.

1. The ‘roller coaster’ of parental emotions.
2. The ‘dark side’ of the unknown.
3. The value of simple pleasures in a life with CF.
4. Reforming clinical care in the new era of CF care.

Each theme will be discussed in detail below. Relevant participant quotations supporting these findings are presented in table 2.

**Table 2:** Main themes and supporting quotes

Main theme	Supporting quote
3.1 The roller coaster of parental emotions	‘When the initially explained to me about it [ETI], I think I was just kind of like, I’m not even thinking that far at the minute. I just wanna get through this neonatal, stay and get out of here before I can even begin to process because they were telling me all about it.’ P3‘We’re getting this life changing medication, This is everything. This is my hope and I would like cling on to and get me through and and that’s it, get me through, and it did get me through.’ P3‘I’m always a bit cautious, I suppose, so hearing about obviously some of the reports on side effects and problems with it, and I follow quite a few of the adults with CF on social media. So obviously hearing that CF, although this drug is, you know fantastic in all that it does, it doesn’t take the cystic fibrosis away.’ P5‘Definitely still want to try you and let’s see how we go, and fingers crossed that just hope that it’s everything I want. I want it to be what I thought it would be when I first heard about it.’ P6‘I don’t know how different we would have reacted to that diagnosis if they hadn’t paired it with the news of ETI I we were already in a complete state of shock.’ P8
3.2 The dark side of the unknown	‘Everyone’s obviously very different, erm, and but I just think for us, and it’s [side effects] frightened us little bit because I think we’ve seen the dark side.’ P3I think you know, once you start it, you can stop if needed. You know, I’m reassured by all that, though.’ P5‘So, since her diagnosis, I have an Instagram account and I follow other mums with kids with CF and I also follow a couple of adults with CF and it’s become more apparent to me about people who ETI hasn’t fully worked for and they haven’t been able to stay on it and I don’t know.’ P8‘I suppose I I am aware, so I am aware of the side effects but to be honest I would rather not think about it too much. I just have an idea of what’s to come, but then choose to focus on it working and picture it working because the thought of it not working is just too terrifying to even let into my head at the moment.’ P4‘It’s a form of control. You do you, you want to be in control. You want to know what? What’s going to happen? And yeah, it’s hard not having control over this situation.’ P6
3.3 The value of simple pleasures in a life with CF	‘You have to do it in the morning (neb) and the Physio and then you have to wash it and then we have to do it at night again and you have to wash it again. People you know wouldn’t know and like you say, it is a simple thing. Yeah, but it is another burden.’ P9‘We’ve been told it should help in a lot of ways, so it can it help reduce the amount of bugs and things they pick up.’ P10‘Yep, it’s really hard and and cancelling plans and so you can’t see people because they’ve got a sniffle, you know, changing Christmas plans last minute, that sort of thing.’ P8‘Well, the life expectancy for me would be, well, my top of the list life expectancy is huge. From whatever is late 20s, whatever we think it is to, you know, 50s plus, you know, that’s massive. That’s huge. So that to me is number 1.’ P4
3.4 Reforming clinical care in the new era of CF care	‘Just want them to live their lives, its not something nice, but and at least it’s only like it it’s only a fingerpick.’ P2‘I have every faith in our team, I feel we’re so fortunate to have, you know, these minds. That and and really do put our faith if they’re telling us this is the right thing, then yeah, That’s it for sure.’ P5‘But you know, so if there was the option just to increase the capacity of the clinic review just temporarily, it would, I think it might be and it might actually help the team because rather than maybe emailing them every week being like all these questions, if I knew I’m seeing them about lesser urgent things in a couple of weeks, I probably would store at my questions rather than ringing all the time about things.’ P5‘We also got and very conflicting advice from and our local hospital doctor and tertiary centre doctor that we see because one of them said that once it’s licence she’ll be on it within a matter of days and, and, one of them said it will be it could be a couple of months.’ P8‘Joey’s [name change] inflamed throat the other day and I wanted to take him up to Open Access at the hospital, but because it’s not directly related to his CF, his breathing, they couldn’t see him. But how do we know going forward what reactions they could have from ETI?’ P9‘Then that you’re gonna have to go sooner and every month or so just for the extra blood tests. You know it’s it’s another added pressure on the normal daily stuff. There is pros in this cons as well.’ P7‘I’m not to look too much online, but I think as a parent, sometimes you can’t help it because it’s only another parent that can relate to you.’ P9

CFcystic fibrosisETIelexacaftor/tezacaftor/ivacaftor

### The roller coaster of parental emotions

**Figure 1:**
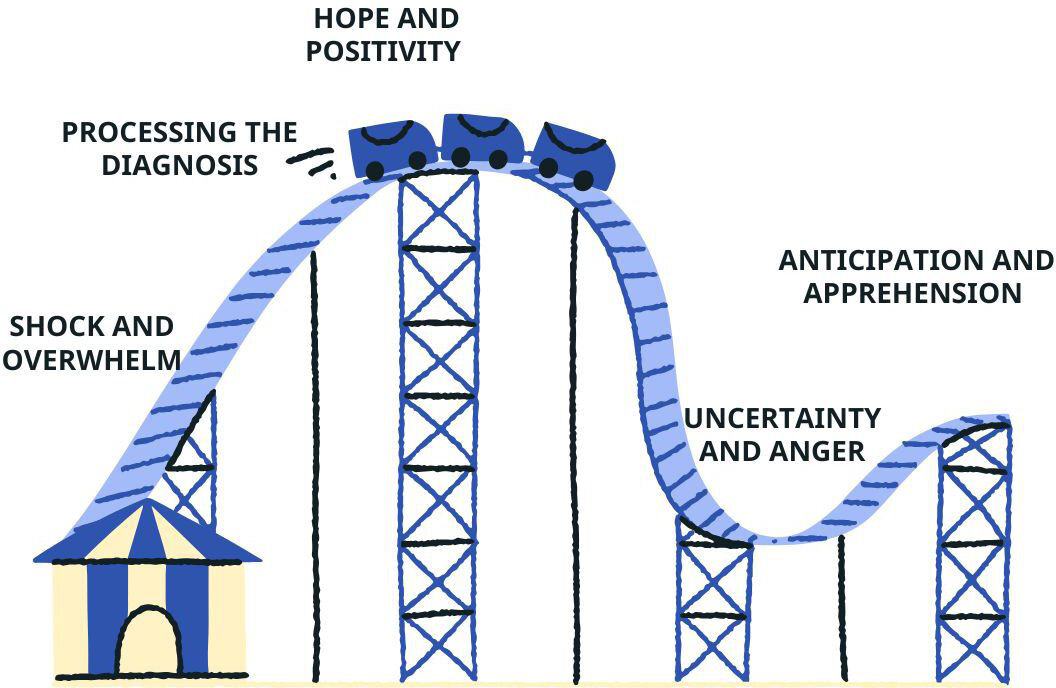
A visual representation of the roller coaster of parental emotions.

The main theme captured was the range of emotions felt by parents from diagnosis. Parents describe the shock and trauma of receiving the diagnosis of CF. Many families heard about ETI during diagnosis which brought hope and positivity. Parents were hopeful that ETI would change their child’s life which made the diagnosis easier to process and gave parents reassurance for the future. Families reported clinicians’ positivity towards ETI and how this brings hope to their child and their future. Following the initial conversations about ETI with clinical teams, parents reported a feeling of anticipation, waiting for the drug. All families then recall a recent setback; the NICE announcement made people think ETI might not be available for young children, with many feelings shocked and angry. One parent reported that they considered ETI a formality and were never told it might not be available. Most participants reflected that as time passed since first hearing about ETI at diagnosis which brought hope, emotions became mixed. Many participants shared that they were nervous and apprehensive about the side effects and the fear that ETI might not work for their child. Most participants, however, reported the desire for their child to start ETI and were hopeful for the future, with one participant reporting, ‘It could be everything we have dreamed of, and it could be our worst nightmare’ P6. Figure 1 shows the emotional responses felt by parents from diagnosis to the present day.

### The dark side of the unknown

All participants were aware of the reported side effects of ETI through social media or clinical teams which caused stress and worry. One parent reported trying not to think about the side effects as the thought of it not working terrified them. On the contrary, two parents wanted to know ‘everything’: every symptom reported and other people’s experiences with ETI; this gave them a greater sense of ‘control’ as so little was known about ETI, a new therapy offered to young children with CF. One parent felt the burden of decision-making regarding whether to start their child on ETI. This parent reported feeling terrified of her child starting ETI due to the side effects of a previous modulator therapy; they reflected on weighing up the physical benefit of ETI against the reported mental-health side effects. They also expressed hope for future treatments with hopefully fewer side effects.

### The value of simple pleasures in a life with CF

A common theme was the desire for ‘normality’ for their child, family, social life and daily routine. Families discussed wanting their child to be ‘normal’ like their friends. The normality extended to other family members, for example, grandparents who also struggle with the treatment burden while babysitting (eg, nebuliser therapy). Treatment burden was a prominent point; many participants reported increasing treatment burden as their child matures and the hope of ETI reducing treatment burden, alongside fewer trips to the hospital, hospital admissions and antibiotics. Parents hoped their child’s future would not be dictated by their health and have a better quality of life; this included reflecting on their child having a job and starting their own family. Risk assessment was a significant part of many participants’ day-to-day routines. Many participants reflected on how ETI might reduce the daily risk assessment and worries around bacteria like Pseudomonas aeruginosa. One participant shared her wish of ‘the simple pleasures of life of taking your kids to a farm, not looking around everywhere and feel, I probably need to do a full-on actual risk assessment’ P3.

### Reforming clinical care in the new era of CF care

Parents expressed gratitude to clinical teams for their ongoing care; some referred to their clinical teams as ‘family’ and felt their children were being closely monitored. Parents endorsed the regular blood tests as they felt it was for the ‘greater good’; ensuring their children tolerated the medication. A desire was expressed to have more frequent contact or clinic visits with the clinical team in the early days of starting new therapies for reassurance. They reflected on how they might not need to see every member of the MDT and how this would reduce the time burden of attending the clinic for monitoring. Parents desired regular and clear communication during the waiting process and after starting ETI. Concerns were raised about how to tell the difference between an ETI-related side effect and a non-ETI-related symptom. One participant reported a desire for healthcare professionals to listen to what parents are telling them, as everyone is still learning about the side effects of this medication. A diverse case was one parent reporting feeling a lack of control regarding making the decision to start her child on ETI; ‘I feel like it is kind of being completely taken out of my hands as to have an option to say, no, I think she is a little bit young at the moment’ P3.

A sense of community was shared and the value of peer support during the era of change and new therapies, as some parents reported being a parent of a child with CF was extremely isolating. They found comfort in knowing that the CF community were all in it together.

## Discussion

This is the first study to explore parents’ views of ETI for young children under the age of 6. Parents reflected on the range of emotions felt in response to becoming aware of new therapies (such as ETI) at diagnosis which is unique for this young age group. Despite the excitement that surrounds ETI, it is an emotionally complex time for the CF community^13^ which is highlighted in the range of emotions felt by parents in this study. Many parents experienced shock during the initial diagnosis. However, the news of ETI brought hope. Hope has been reported as a prominent experience of adults taking ETI; hope for the future due to improvements in physical health.^14 15^ Over time, mixed emotions of uncertainty and apprehension regarding the medication were felt by many. Parental apprehension has also been reported by families of adolescents with CF, worried if the long-term benefit of ETI will be sustained.^2^ Parents, however, reported waiting in anticipation for ETI and remain hopeful for the future. Supporting healthy parental adjustment is essential in infancy as this can impact the child’s quality of life and family, treatment adherence and engagement with the CF team.^6 14^

All parents expressed knowledge of reported ETI side effects which led to parental stress and anxiety. Worries about access to the medication in the future were felt among many parents, as they were concerned that their child might have to discontinue ETI. The worry regarding discontinuation aligns with the adult population, where some report living in fear of ETI discontinuation due to other health complications, reporting that returning to life pre-ETI was unimaginable.^15^ We must support parents with the diagnosis of CF, stress and positive family functioning^16^ and now that new therapies are becoming available.

Many parents hope to simplify the treatment burden, positively impacting their family life. Prior to ETI, many caregivers and pwCF reported burnout from doing treatments every day.^3 17^ Many parents also expressed their hope for an improved quality of life for their children and for the future not to be dictated by their health. Positively, adults who commenced ETI felt more opportunities in life, as CF did not play such a critical role in their lives. This has also been reported by parents of adolescents with CF, reporting the positive impact of modulator therapy on the quality of life and diminishing treatment burden.^2^ Parents reflected on the daily risk assessment and worries regarding pathogens. Before ETI, many parents of children with CF have been concerned about acquiring pathogens like Pseudomonas aeruginosa from the environment and placed many restrictions on domiciliary and outdoor surroundings and activities, as discussed by participants from this study.^18^

Many participants also reflected on how the NICE statement impacted the CF community in the UK, causing worry regarding future access to ETI. Many participants felt supported by the clinical team and accepted the additional investigations to ensure the safety of the medication. Families reflected on the type of support they deemed necessary in the early days of starting ETI (or new therapies) and encouraged clinicians to consider reforming care for those with CF eligible for modulator therapy. A recent study reflected on the careful thought clinicians must give to conversations around modulator therapy focusing on ongoing side effects, disease‐specific and health‐specific goals and broader life goals.^9^ Examples of how care teams could best support patients and their families are;

- Psychological support for parents of young children with CF, especially during the introduction of new therapies.
- Clinicians should consider the role of parental peer support.
- Teams should consider the clinical contact and capacity, which may be required following the introduction of new medication, to ensure parental reassurance.
- Regular and clear communication between families and the clinical team.
- Reflect on routine clinical appointment length in the long term.
- Dietitian advice and education about symptoms to look out for when starting ETI; ideas regarding a variety of food for fussy eaters; offering information on food ideas for young children taking ETI.
- Support and listening to help distinguish side effects and behaviours in children that are ETI and non-ETI related.
- Families want healthcare professionals to listen to them and wish for joint decision-making as new therapies become available to younger children.

This study is a single-centre study (UK) and homogeneity is related to sex, ethnicity and race (all participants interviewed were British and primarily female) which is a limitation of this study. The perceptions of ETI were based on the lived experiences of a small sample size; a larger sample size would strengthen the credibility of the findings and data saturation. A strength of this study was that all focus groups were conducted by a female physiotherapist (BSc) interested in qualitative research methods, who also underwent qualitative training with the research team. As the research team worked closely with the participants, it felt important that the data collector had no experience in CF to reduce prior assumptions during the data collection and to encourage open conversations with participants. Important aspects of the study have been reported in line with the Consolidated Criteria for Reporting Qualitative Research checklist and is one of the first studies on parents’ perceptions before the introduction of ETI for young children.

## Conclusion

Parents of young children eligible for ETI experience a range of emotions while their hope for their child’s future is clear. Teams must communicate and acknowledge the anxieties regarding new therapies and anticipate the support families require when introducing new therapies. Further research is needed to understand better the long-term impact of modulator therapy on the mental health and coping strategies of parents and caregivers.

## supplementary material

10.1136/bmjresp-2024-002522 online supplemental appendix 1

10.1136/bmjresp-2024-002522 online supplemental material 1

## Data Availability

All data relevant to the study are included in the article or uploaded as supplementary information.
