# Supplementary material for: Qualitative study exploring the views and perceptions of parents/carers of young children with CF regarding the introduction of CFTR modulator therapy (The REVEAL study; PaRents pErspectiVEs of KAftrio in chiLdren aged 2–5)
Source: BMJ Open Respir Res. 2025 Jan 30;12(1):e002522. doi: 10.1136/bmjresp-2024-002522 (PMC11784110; doi:10.1136/bmjresp-2024-002522)
Supplement: online supplemental appendix 1 [file bmjresp-12-1-s001.pdf]

Appendix 1

FOCUS GROUP TOPIC GUIDE

Research goals of the focus groups;

§ Perception of the medication and how best to support families and their children as they move onto new, potentially life changing medications.

THE FIRST BLOCK OF THE FOCUS GROUP

1. Introduction, overview of focus group and objectives – this takes the first 25 min. (approximately):

· Welcome, introduction of researchers and the project

· Instructions regarding the focus group: *"We are interested in how parents/ guardians feel before the introduction of Kaftrio and what support might be beneficial from the clinical team"*. *We will discuss feelings; expectations of the medication and any realities [practical considerations] people are thinking about before the medication becomes available.*

· Instructions*: "Please be assured there are no right or wrong answer to the questions, this is a safe and confidential environment. Please can I encourage everyone not to discuss the content of this focus group outside of this environment to respect everyone's privacy. If you wish not to use your child/children's name in this discussion you do not have to.*

The interviewer's job is to ask questions, manage time and move on if necessary. and one person taking notes. The focus group will take approximately 60 minutes.

People will have very different experiences and feelings within this group, please be assured your own personal views matter.

If you wish to withdraw you are free to do so [and it will not affect you or your child's care] up until the end of this focus group. We are unable to do so after as this conversation will be typed and anonymised.

Any thoughts/ worries from this focus group please discuss with the clinical team who look after your child/ children. Please try not to compare a story/ child to your situation as every child is an individual. We will let you know when we are coming near the end of the focus group and if people are happy to share What they think are the key message we have captured within this group."

Intro/ warm up; *"Please introduce yourself and the age of the child (if you are happy to) and share a recent example that made you smile with/ about your child/ children [as children are at the heart of the work we do.]"*

Detailed exploration

- **Perceptions** ; Parents to share their feeling/ thoughts surrounding kaftrio.

"Can anyone share when they first heard about Kaftrio and how you felt?"

The researcher notes down activities mentioned by the parents/ guardians and will talk them over later. The researcher structures the activities thematically. Please expand on situations/ feelings mentioned, example given below.

e.g some of you mentioned feelings waiting for the medication to become available, are others happy to share their feelings

e.g Some people mentioned social media, can you share your experiences?

e.g people have mentioned the medication being mentioned around diagnosis, what did this make you feel?

e.g anyone else- can you describe any feelings you had in the early days or now about kaftrio?

These questions can be discussed for quite a long time, about 20 minutes, so that the families can discuss feelings.

At this point they may mention some **“expectations” of the medications** and worries attached to this -> the research will note down the topics on a piece of paper – and discuss them later.

If the parents discuss a worry spontaneously, we do not interfere.

Focusing on key areas

2. The following 20 minutes (approximately)

- _We inquire about the expectations parents have for the medication.; Some people have mentioned hope.. What would you say your hopes/ expectations are for your child starting kaftrio”_
- _E.g Realities; Some people mentioned side effects or blood tests prior to the medication. “Is there anything on your mind/ any worries in regard to kaftrio”?_
- Note down any feelings or expectations discussed.
- Spontaneous discussion is welcomed.
- We can ask about practical points in more details e.g what side effects have you heard of any was this through clinicians/ people you know?

1.How do you feel about your child having a blood test to monitor the medication?

2. E._g some people mentioned *****, what support do you feel you would like during this journey?_
3. _A few people have mentioned the **** feeling, how might people support you with this feeling?_
4. E.g some people mentioned hope for the future, what other feelings do you have if we focus on the future?

- _“Do you have any questions to be feed back to the team about Kaftrio?”_
  - _“Can you think of anything else? Please be assured every question and thought is important. “_

- It may happen that a parent/ guardian will mention a very specific thing e.g do you know how much benefit a 2-year-old has from this medication, please be clear that as a research facilitator we do not know but this can be given to the team as a question to address if people consent?

Summarising and reflecting

“We are coming to the end of the session; we will reflect on today’s discussion and summarise the main points.”

· Interviewer to sum up what has been discussed, mention the feelings, perception of the medication and realities.

- “*Would people be happy to share what they have found most relevant or important from today’s discussion?”*

Conclusion – about 10 minutes:

“Thank you for taking the time to participate in this discussion, is there anything important to you we haven't mentioned? If you want to follow any issues you have talked about, you can contact the clinical team via telephone or email.”
