# Supplementary material for: Qualitative study exploring the views and perceptions of parents/carers of young children with CF regarding the introduction of CFTR modulator therapy (The REVEAL study; PaRents pErspectiVEs of KAftrio in chiLdren aged 2–5)
Source: BMJ Open Respir Res. 2025 Jan 30;12(1):e002522. doi: 10.1136/bmjresp-2024-002522 (PMC11784110; doi:10.1136/bmjresp-2024-002522)
Supplement: online supplemental material 1 [file bmjresp-12-1-s002.pdf]

Theme	Code description	Relevant quotes	Notes/ Key dimensions to participants responses to report in the final paper

**The "roller coaster" of parental emotions**	Range of feeling felt from initial diagnosis to present time.<br><br>Turbulent journey from shock/trauma to hope/ positivity.<br><br>Some parents feeling different about it now. Uncertainty about the future, realism.<br><br>Anticipation and wanting to try Kaftrio despite concerns re side effects<br><br>Uncertainty regarding future funding/ access to Kaftrio.	It was our first meeting with the consultant. He mentioned Kaftrio and obviously cause well, we like well, personally, like when I we got the news, I wasn't really like processing kaftrio at all because it was all very quite overwhelming. P1<br><br>Having his diagnosis so, because that was so overwhelming, I I didn't really like fully understand at that moment how amazing this modulator is. So, but then obviously like with time like after that, we've like obviously found out a lot more about it and stuff and they've discussed it. P1<br><br>basically I would remember them saying like obviously it's not good news with the diagnosis, but if there's ever a time, to have CF it's now because because of this drug. P2<br><br>Definitely like hope, but I think at that moment it was just so overwhelming .P1<br><br>So you see all like the life expectancies people on CF and like that was a big shock, but then they're saying now obviously that's just gonna get like smashed out the window, which is for us all, for me it was obviously it's exciting that he's gonna be on. It's like less of a worry for his future. P2<br><br>So I think we've just been really really excited for it to, you know, and I think like we won't believe it until it's in our hands now. P2<br><br>But like after that initial diagnosis and everything like that, definitely like hope and a lot more like positivity and hmm, yeah, definitely. P1<br><br>I was just trying to approach of what her life was going to look like P3<br><br>When she first got, like, officially diagnosed, it's like, you know, this drugs coming and and like, you know, I will be hopefully one day change your life and stuff like that. P3<br><br>It's hard because like along with it, the diagnosis came the news of like kaftrio. So it was hard to like have this like, elated feeling, which maybe like other people might feel like. P1<br><br>But you still got so many questions yourself about the actual condition. So you're asking like those questions about the condition that you trying to process that, but definitely like made it feel, you know, and especially because then you have so many people saying, but there's this	Overwhelming diagnosis<br><br>Overwhelming diagnosis<br><br>Hope<br><br>Hope/ overwhelming<br><br>Shock initial – reassuring<br><br>Positivity/ Hope<br><br>Hope/ positivity<br><br>Uncertainty for the future<br><br>Positivity<br><br>Processing<br><br>Processing

		amazing drug, there's this, you know and over time that definitely means everything so much easier to process. P1<br><br>When the initially explained to me about it [kaftrio], I think I was just kind of like, I'm not even thinking that far at the minute. I just wanna get through this neonatal, stay and get out of here before I can even begin to process because they were telling me all about it. P3<br><br>I just felt so overwhelmed with the situation I was in at the time. P3<br><br>Because I think obviously, like we had a lot to process at the time, which just diagnosis alone. But like over time like or, it's just been so, Just waiting for the drug, Really. P1<br><br>We've even, like said like all you can start nursery maybe went like we want them to have the kaftrio. So like a lot of things, we've just kind of been like hoping and waiting for this.P1<br><br>Nice news the few weeks ago and really like settles back, didn't it? P1<br><br>Yeah, I think more because we've never been told It was never gonna be not available. Really, that was more of a shock to the system P2<br><br>remember asking when he first got diagnosed and he mentioned kaftrio, remember mentioning like a warranty? **Like what is they up the** prices cause you hear about all the time, don't you? P2<br><br>We were from to have it and like hopeful and you know, think like it's future as like looking really bright and stuff. So to that, just kind of really settles back. P1<br><br>Like had I've not really looked forward to a start because I'm terrified of what if it happens again and where does that leave us, if she can't tolerate it because that's two drugs shes been eligible for that she can't tolerate P3<br><br>Or is he gonna be something else in the pipeline now? Could maybe treat her maybe that's a bit more better for her body type of thing P3<br><br>I guess we've just got to for , think it's like it I feel like would I'd be apprehensive starting something new, but we shall wait and see. P1	Processing<br><br>Overwhelmed<br><br>Processing/ anticipation<br><br>Waiting<br><br>Shock<br><br>Shock<br><br>Uncertainty regarding availability of kaftrio<br><br>Uncertainty<br><br>Terrified if they do not tolerate<br><br>Hopeful for the future<br><br>Apprehensive, give it a go<br><br>Trauma initial diagnosis

		She was about four months and the first four months were just so traumatic. P4<br><br>We're getting this life changing medication, This is everything. This is my hope and I would like cling on to and get me through and and that's it, get me through, and it did get me through P4<br><br>I remember literally within that conversation [diagnosis], Kaftrio was brought up as in, you know, yes, there's this, you know, life limiting diagnosis, but you know, there is this drug coming down the line, I think it's been like a a countdown, a countdown. When do we get to that? You know every whatever, every infection, every birthday, every step is 1 little bit closer to this life changing drug that I think that we all hope and pray works the way we, we dream it's gonna work. P4<br><br>I'm always a bit cautious, I suppose, so hearing about obviously some of the reports on side effects and problems with it, and I follow quite a few of the adults with CF on social media. So obviously hearing that CF, although this drug is, you know fantastic in all that it does, it doesn't take the cystic fibrosis away. P5.<br><br>I will remain anxious as a mummy I think forever, regardless of any medications that come up, I don't think it's going to change everything. P5<br><br>I think when she, when, when, we were told about kaftrio. I was just like this is going to work no matter what it's going to be amazing. P6<br><br>But wasnt expecting her liver to be the way it, it was on her results, and it’s just throwntaken everything for me with Kaftrio, because I know one of the side effects can be liver issues, and you know my main concern is, OK, what if it doesn't work? What if she can't go on it, and that's what's kept me going, Kaftrio, now I think everything's 50/50 for us right now. P6<br><br>I feel so different about it now compared to how it did when she was diagnosed. P6<br><br>But we are staying hopeful and I how people with liver issues and you know liver disease and do an OK on it. But it's just the what if? P6<br><br>definitely still want to try you and let's see how we go, and fingers crossed that just hope that it's everything I want. I want it to be what I thought it would be when I first heard about it. P6	Hope<br><br>Anticipation /Hope<br><br>Cautious<br><br>Parental anxiety<br><br>Hope<br><br>Concern due to side effects/ not working<br><br>Change in feelings<br><br>Hopeful / what if it doesnt work<br><br>Want to try<br><br>Terrified- might not tolerate it / No hope

		But you know, there is always that massive risk that he'll have to be taken off kaftrio because he's livers working on it, and that's the terrifying thing, isn't it? You think that this amazing drug is gonna save them, but it could make them so much worse. And then you feel like you're back to square one without the, without the hope. P4<br><br>I don't see it is a decision. I think it's a there isn't a decision, it's an absolute yes. And and you. And if it doesn't work then it doesn't work. I don't think it's. It's never been a decision whether we try it or not. P4<br><br>I'd say no matter what we do, it's no question we, we wanna give it a go. It could be everything we've dreamed of and it could be our worst nightmare. We we just don't know, but we just wanna be given that chance to go on it and see and see how we go. P6<br><br>There's not been much information around these latest worrying developments, you know about the approval and all that and that lack of information has been difficult. P5<br><br>But I feel I'm a bit angry that that wasn't, you know, we weren't made aware of this. I suppose I don't know. Angry at who? I'm not directing that at anyone in particular? But just angry that we weren't aware that that process P5<br><br>There was no care or thought or anything to how that information came out, and there were some very, very terrified families across the the country. P4<br><br>This is a drug that gives our children a future, so to not have that and to have no reassurance or you know, whatever it might be is a scary place. P4<br><br>I think the other big thing might be others have said like it's the .. It's the heightened anxiety that you live on every day. You know the anxiety........ one second, the anxiety is is always there. Like it. It never goes away. P4<br><br>So we are going through the play specialist and through that so and it's not gonna be very nice, but it sounds awful for me to say, but if that's what it takes for it to get a drug, that's, that's what we have to go through and we just have to, you know, make it the best of what we can. P4<br><br>I think it's as soon as they go on it, we're gonna be watching them like a hawk you know, every single sign symptom we're gonna be watching them 24/7 P6	Want to try<br><br>Want to try<br><br>Worry regarding funding<br><br>Anger following NICE process<br><br>Terrified families- NICE process<br><br>Scared parents – NICE process<br><br>Parental anxiety<br><br>Endevour<br><br>Parental anxiety<br><br>Frustration<br><br>Uncertainty

		I don't understand why the team haven't got any urgency P5	Traumatic – NICE
		I don't trust the government and we I want the prescription on his record that he is on this. If vertex are gonna mess about, not because they haven't got the label for the box or I'm gonna supply for this dose or whatever, why can they still not do the prescription? It's they're able to prescribe it now as per the MHRA, it's licensed, Just prescribe it. P5	Parental stress
		This is a dream drug we've been promised for years and years. And you know, up until a couple of months ago, a month ago, it was almost taken away from us. So this is something that you know, we we don't forget about as parents. We're thinking about all day, every day at the back of our mind. P1	Anticipation from diagnosis<br><br>Hope
		You know, as soon as we know just I can they they have said they'll tell us when they know, but we're all on pins. So I think my ability to remain rational is gone. P5	Anticipation
		it was literally I had been released and they, they were bringing it down, the age categories already, and so when she was born, they said that they would anticipate by the time she was two that it would be on the cards for her to take. P8	
		And so yeah, light at the end of the tunnel, I think. P8	Reassurance
		So they were like we know lots about this and there's a medicine out there already, that was literally like in the first sentence or two of them giving her the diagnosis, so I think from the very off it's been, ok, come on, she needs to get on this drug so I don't know, just a feeling and sort of anticipating it. P8	Reassurance<br><br>Shock – diagnosis
		There's been a lot of reassurance come from it. And like when we got the diagnosis and told people they'd be like ….. so and so's got that and you know, it's a lot better than it was years ago, and so when he said about the new drug, you know it's it is, it's you feel like there's, you know it's not it's not gonna be the end of the world type of thing. P7	Shattered with diagnosis – Hope<br><br>Anticipation
		It is reassuring that there's better solutions out there for them and they'll get a better life out of it. P7	Mixed emotions
		I don't know how different we would have reacted to that diagnosis if they hadn't paired it with the news of kaftrio I we were already in a complete state of shock. P8	Positivity

		That first year obviously shattered with the diagnosis, but then it gives you a little bit of hope. P9<br><br>And then I think your just on the edge of your seat then waiting and waiting and waiting, but nothing. P9<br><br>Now the times near, I don't know. It's kind of mixed emotions because of everybody calls it a wonder drug, but until the child actually starts that you don't know how they're gonna react to. P9<br><br>There's a lot of positivity around about the drug coming. P7<br><br>I, I'd say that I'm still very hopeful and I mean it's been you know, top of the sort of erm conversation so like. P8<br><br>I think the reality of what if it doesn't work and it might not work for Cassy and that's just sort of, I don't know, it's like it's got the reigns on my emotions a little bit to not get carried away. P8<br><br>When I was first told about it at the diagnosis, it was I'd say it was more a this will work, this will change her life. P8<br><br>I don't know. I just sort of trying not to get my hopes up too much, whereas still being really, really anxious for her, to be honest, it's difficult to manage your feelings actually I think, about it. P8<br><br>I feel quite apprehensive about it and just the wait, and whether I should say whether it will work or what, what, what size effects is it gonna come with because at the minute like John looks so healthy and on the outside looks OK but still suffers his problems. So, it's, you know, whether it'll take that away or whether you know it might get worse side affect. So yeah, it's quite nervous. P7<br><br>Just that obviously wanna get him on it as soon as possible; And that, like, say, apart from the side effects like so just want to see how it goes obviously because obviously there is side effects and obviously different doses you can get as well and things like that so. P10<br><br>No one else can relate to to the no one else can relate to the trauma, to be completely transparent about it all. It's an enormous trauma. I was just saying before, we had no idea that she had meconium ileus until she was born. P8	Hopeful<br><br>Reign on emotions<br><br>Positivity<br><br>Parental anxiety<br><br>Apprehension<br><br>Anticipation<br><br>Traumatic (initial)<br><br>Parental anxiety<br><br>Realism<br><br>Uncertainty

		Obviously, it's anxiety and the lead up to it [procedure]. P9<br><br>I think we have to be realistic; We know it's it's not gonna cure. P9<br><br>But we haven't got a clue. I I've never been in this position before with a new drug. P8	

The dark side of the unknown;	Frightened of side effects<br><br>Burden of decision making<br><br>Social media reports<br><br>Control (information seeking) vs reducing exposure to the what if<br><br>Range of side effects reported by all parent<br><br>One parent traumatised after side effects of different medication	And she was in considerable for an entire week to the point where I had to phone because from one day and get him to come home from work when I was on leave because I couldn't cope anymore. P3<br><br>She clearly can't tolerate it, erm so ever since then I've been a bit traumatized about kaftrio. P3<br><br>Everyone's obviously very different erm and but I just think for us, and it's [side effects] frightened us little bit because I think we've seen the dark side. P3<br><br>I am terrified. I'm absolutely traumatized by what happened with orkambi. P3<br><br>I've got that worry like that burning worry if the back of my mind if she doesn't tolerate it again P3<br><br>It's the neurological side effects I am and I think it's the feeling of having to choose between our physical well being and a mental well being. And I just think other parents how am I meant to make that choice and and then I've seen a lot of like, obviously adults who were on kaftrio and it's become a more and more prevalent now that they're coming out and saying I have got really bad mental health issues since starting kaftrio now and I've got insomnia. P3<br><br>Think of feeling like that choice that burden of choice is about to be landed on may me about physical or mental well being, and I just don't know. P3<br><br>I've like read a lot about it, and I've heard that that's like a like a lot of the side effects can be to do with, like, mental well being and things and obviously like an and yeah, that is probably again the worry umm. And but we've not had the experience yet. So, but that's probably the worry in the back of my mind. P1<br><br>They [adults on social media] said there was like a fog over the brain and they cant make a decision. Maybe not as sharp as what they used to P3<br><br>I think you know, once you start it, you can stop if needed. You know, I'm reassured by all that, though. P5	Parental stress<br><br>Trauma- following side effects<br><br>Frightened of side effects<br><br>Trauma- following side effects<br><br>Parental anxiety following side effects<br><br>Burden of decision making<br><br>Burden of decision making<br><br>Burden of decision making<br><br>Worry / Social media reports<br><br>Reassured can be stopped

		And I think why put myself through that if I don't need to? Don't need to do it, so for now. I am aware and you listen to some bits, but I would rather not research because it might not happen. P4<br><br>I suppose I I am aware, so I am aware of the side effects but to be honest I would rather not think about it too much. I just have an idea of what's to come, but then choose to focus on it working and picture it working because the thought of it not working is just too terrifying to even let into my head at the moment. P4<br><br>I feel like I need all the info and I don't know. It's probably not a good thing. I think it helps me feel I'm a bit more in control in a way I don't in life in general, I don't like not having control. P5<br><br>I wanna know everything. I wanna know every possible outcome on Kaftrio. I wanna know everybody's story. I wanna know every single symptom. I wanna know exactly what I'm looking for. P6<br><br>It's a form of control. You do you, you want to be in control. You want to know what? What's going to happen? And yeah, it's hard not having control over this situation. P6<br><br>And one of the CF nurses said it's a mild form of CF related liver disease, and we're gonna monitor it and see how she goes. And I just felt sick because my first thought was kaftrio. P6<br><br>There's so much more awareness and it's about it now, So many more people coming forward about the side effects and I know a lot of children are suffering from a behaviour changes. P6<br><br>I think for me the biggest thing is is the liver and I think it's for the others as well as well because that is a game over. If it's not working, you know it's as simple as that. P4<br><br>I've heard from others hyperactivity isa thing and it doesn't sound like a massive thing, but if your childrens all are already got ADHD as well and you put them in school situations when they're already struggling to learn, throw in extra hyperactivity as well. P4<br><br>Also the sleep, the the insomnia and the the moods changes. And you know from what I've heard from others, it's like, you know, very different children one day to the. P4<br><br>The personality change I've heard that yeah. And and again I mean that's like upsetting to know your child fully and then after a couple of weeks, you've got a completely different child. P6	Choosing to limit exposure to side effect reports<br><br>Terrified/ Hopeful<br><br>Information gathering<br><br>Control<br><br>Information seeking<br><br>Control<br><br>Parental worry/ concern<br><br>Increased reports of side effects / Liver complications<br><br>Impacting learning/ behaviour<br><br>Sleep, mood<br><br>Personality change<br><br>Hope they don't have to stop it

		And I hope that decision is not doesn't have to be there that, you know, do you have to take them off. P1<br><br>I have no faith in the government, and I pessimistically worry about the future of the NHS and then that what means what that means for people with CF and loads of the chronic diseases and you wouldn't get health insurance and all the rest of it so. P5<br><br>So since her diagnosis, I have an Instagram account and I follow other mums with kids with CF and I also follow a couple of adults with CF and it's become more apparent to me about people who kaftrio hasn't fully worked for and they haven't been able to stay on it and I don't know. P8<br><br>Yeah, I'm at follow quite a lot of people on social media, Instagram now and in the beginning I wouldn't have even uttered it, I thought. But as you people, you do say, obviously, mental health creeps in as a big sad, change. And so I'm apprehensive about that cause because of the age. Don't know, he's got such a cracking personality and lovable and funny. And it's that worry that, that could change. P9<br><br>I'm more concerned with the liver side effects because she was on TPN for an awful long time and that damaged the liver quite a lot. And we've only just the her last annual review, when she was two, got down to normal number. P8<br><br>For her liver enzymes, so, and I know that that's a big thing that they wanna keep an eye on is a liver. So that's my, I would say primary concern. P8<br><br>I know there's the mental health side of it as well. And when they if they get headaches and that sort of thing P8<br><br>Especially mental health could because it's bad enough as an adult having mental health. So someone so young to have to suffer, you know, it's quite scary. P7	Worry about future access<br><br>Social media reports<br><br>Social media repors – mental health<br><br>Liver side effects<br><br>Liver primary concern<br><br>Mental health/ headaches<br><br>Mental health- scared parent
The value of simple pleasures in a life with CF;	Reduce treatment burden<br><br>Normality for the child and support	When you think about him having children and stuff like will that be able to happen?<br>You, you know, and everything just feels a bit more hopeful now. P1<br><br>Like I think obviously like my kinda like things is if she does tolerate it as a gonna lessen the treatment burden. The little bit, because she's being on nebulizer since she was a couple of months old. P3	Future- family<br><br>Reduce treatment burden

	network (e.g extended family)<br><br>Thinking of the future<br><br>Reduce risk assessment need and result anxiety<br><br>Parental demand/ sacrifice	Will that lessen that burdent a little bit and we can maybe come off even at least one of the nebulisers or one of the inhalers like if we can just drop one thing out of her day I feel like it makes so much of a difference rather than the daily battle of trying to reason with her about why she's gotta do these treatments day in day out. P3<br><br>So she can be a normal kid, like every like all of the friends. P3<br><br>I think we just like just lead a normal life and I hope that it's quality of life is like is good and we don't like have that well especially me have that anxiety when we're like we're in places where I feel like I have to risk assess everything rather than just let him be a child. P1<br><br>Just hoping like he can, like have a job and, like, have a family and be happy. And that like see CF doesnt take over his life. P1<br><br>Doesn't stop him from doing things. P1<br><br>Hopefully it will mean if she's ok on it, less trips to hospital last admission and less antibiotics because she just seems to be constantly on them at the minute with cough and cold season P3<br><br>About the risk assessment, like to just be able to go to like, I don't know, like a farm and not sit there like the simple pleasures and life of taking your kids to a farm, not looking around everywhere and feel I have probably need to do a of a full-on actual risk assessments. P3<br><br>Obviously, I want her to have that choice and that opportunity and her health no to dictate, P3<br><br>And I think you just worried about all kinds of mental trauma. is that gonna leave on them getting poked and prodded all the time. P3	Reduce treatment burden<br><br>Normality – child<br><br>Future- quality of life / risk assessment<br><br>Future not dictated by health<br><br>Future not dictated by health<br><br>Reduce burden- less hospital trips<br><br>Risk assessment ; farm<br><br>Future not dictated by health- choice and opportunity<br><br>Long term impact intervention<br><br>Treatment burden – physio

		The treatment burdens have gone up so much, to, you know, nebulisers like four times a day. Physio 3 a lot of lot more medication in between so I think this is one of the things we spoke about with our team is to just try and but hopefully you know cut down on some of these treatments. P6<br><br>She so upset at the moment we feel like we can't get out some days because of the time of the nebulisers and the Physio we feel like we can't meet up with our friends and making a specific time to meet up somewhere. P6<br><br>As well as like treatment burden, I can hope for that. Maybe infections won't linger or whatever, in the same way. P5<br><br>If we can tolerate this drug, the you know all these other things don't become a scary as what they are. You know, it would be lovely, wouldn't it? For he does play rugby, but you know for him, me not to feel terrified of the mud on his face. P5<br><br>The first thought not to be like crap like and be thinking about Pseudomonas if things like that. P5<br><br>You know for the CF to be less present in every day. P5<br><br>So even my who I want to leave him with. And you know, because they don't do it as good as I feel I can do it. And because it because the CF isn't at the forefront of their mind and nor, nor do I want it to be. P5<br><br>Of yeah, infections and stuff. P6<br><br>Well, the life expectancy for me would be, well, my top of the list life expectancy is huge. From whatever is late 20s, whatever we think it is to, you know, 50s plus, you know, that's massive. That's huge. So that to me is number 1. P4<br><br>If Kaftrio can help Noah's belly, that that's massive because you know, it's a difference between going to the toilet eight times a day to going once a day. P4	Treatment burden impacting social life<br><br>Less infections<br><br>Risk assessment ; mud<br><br>Worries around pathogens<br><br>CF to be less present in everyday<br><br>Support network<br><br>Less infections<br><br>Life expectancy<br><br>Quality of life- bowels<br><br>Reduction parents anxiety<br><br>Parental sacrifice

		So to have a little slice of that [anxiety] taken away and to be a bit less neurotic mother would be would be really nice not to worry so much. P4<br><br>The logistics of having to go another burden, you know, having to miss school. I I've organised my whole working week around not working on a Wednesday to so I can always be available to go for clinic cause over my dead body. P5<br><br>Whatever it is on my mind, until I know that it's signed off so it it will be another, you know thing every time. And that's probably more the worry than the logistics than the actual physical test. It's the the waiting for the results. P5<br><br>Yeah, it's more so the waiting. It'd be the waiting for the test, the anxiety of waiting. We feel that anyway, after each clinic, after each cough swab. P6<br><br>Yep, it's really hard and and cancelling plans and so you can't see people because they've got a sniffle, you know, changing Christmas plans last minute, that sort of thing. P8<br><br>Yeah, as much as you are told to, don't let it rule your life and have a normal life. It it does massively impact it. P9<br><br>We’ve been told it should help in a lot of ways, so it can it help reduce the amount of bugs and things they pick up. P10<br><br>Yeah, but we're just keen, obviously, Joey, due to start school in September. So keen to get him established on it and see how he is before then, like they said before the the next winter kicks in as well and. P10<br><br>I'd really love to get rid of the nebulizer; I hate washing that thing up every day. I'd love to get rid of that. P8<br><br>Yeah, yeah, it seems like the simple thing, doesn't it? P9<br><br>You have to do it in the morning and the Physio and then you have to wash it and then we have to do it at night again and you have to wash it again. People you know wouldn't know and like you say, it is a simple thing. Yeah, but it is another burden. P9	Waiting for results<br><br>Waiting for results<br><br>Impact on family life<br><br>Impact on family life<br><br>Reduce infections<br><br>Reduce infections<br><br>Reduce treatment burden<br><br>Simple things<br><br>Treatment burden- physio<br><br>Impact on family life<br><br>Support network<br><br>Treatment burden

		If you want to be out for tea and it's that bit later, well, you you rushing home because you gotta get these other things done before, just the norm bedtime stuff. P9<br><br>Yeah, and yeah, if my parents are looking after her in the evening and putting her to bed, though, if I'm at work, then she ends up like my mom hates doing the nebuliser, she just gets really stressed with it, and it'd be nice to to not have to leave her with that to, to deal with. I would love to get rid of popping pills and stuff. I think is just it's, It's, That's not a big deal for me. P8<br><br>So it's not like, ohh, I'tll let me take a minute. You know it's 7 minutes (nebuliser) or it could take longer if the refusing to do it and then before you know it, it's like an hours gone by and you still haven't go P7<br><br>Like I is said about the nebulizers and stuff, if we can get to a point where actually just her daily routine is more similar to a normal toddler than having to have all the these extra interventions and having to write a list down for my parents of things that she has to take if I leave her with them for a few hours and and if in a a year or two we can chill out a bit more about going to Chester Zoo. So she can go and the orangutan house because it's got water misters in there and you know that and all The Walking through the Butterfly House. Just things like that would be. Really nice to just even consider or at, at the moment it's just a hard no. P8<br><br>This is your appointment time or I mean she literally had a blood test done yesterday and my mom had to take her because I'm at work and I can't take more time off work. P8<br><br>I did go back to work after mat leave from work from home because it was covid. But then he was in hospital so much, I had to end up leaving my job. I've been there for 11 years, so that is a massive hole that's left in me. My life had to completely change, but. And most people don't experience in that when you've had, had a child. They just go back to work as normal and it's fine for them to just go straight into childcare. Or so whatever you choose. P9	Treatment burden/ support network/ risk assessment<br><br>Parental demand<br><br>Parental sacrifice
**Reforming clinical care in the new era of CF care**	Joint decision making?<br><br>Appreciation of the clinical team<br><br>Clinic practicalities;	I do feel like there's a certain degree of pressure from the clinical teams. P3<br><br>I feel like it's kind of being completely taken out of my hands as to after option to say “No, I think she's a little bit young at the moment, can we just maybe wait until she's a little bit bigger? P3<br><br>I always feel they like always feel like the they’re monitoring us like so anything like changes in outpatients. P1	Pressure<br><br>Decision making<br><br>Monitoring

	frequency, length, staffing<br><br>Reassurance and support for families<br><br>Communication<br><br>Peer-support and community<br><br>Isolation as a parent	Any changes they we just tell them and they'd support us with whatever anyway. P1<br><br>Just want them to live their lives, its not something nice, but and at least it's only like it it's only a fingerprick. P2<br><br>Yeah, or but at the end of the day, for the grater are good, isn't it? If it's gonna, yeah, be beneficial. P2<br><br>I have every faith in our team at ***, I feel we're so fortunate to have, you know, these minds. That and and really do put our faith if they're telling us this is the right thing, then yeah, That's it for sure P5<br><br>Psychologist on speed dial would be mine. P4<br><br>I think some facilitated groups like this with a clinician on would probably be good 'cause I imagine we'll probably all have the same as this just come out in this. We've all got the same worries. They're not, You know, we've all got our own opinions and things, but a lot of the questions and anxieties are all gonna be the same. P5<br><br>Instead of messaging the team every 5 minutes, I just think it'd be nice like once a week if there's like a support group something like this, we can more jump on. We can all have our questions ready, you know? P6<br><br>More frequent, just to say perhaps more frequent clinics. P5<br><br>But you know, so if there was the option just to increase the capacity of the clinic review just temporarily, it would, I think it might be and it might actually help the team because rather than maybe emailing them every week being like all these questions, if I knew I'm seeing them about lesser urgent things in a couple of weeks, I probably would store at my questions rather than	Good support<br><br>Regular monitoring endorsement<br><br>Regular monitoring endorsement<br><br>Trust in clinical team<br><br>Psychology support<br><br>Peer-support/clinician groups<br><br>Support group<br><br>Clinic frequency<br><br>Clinical review/ frequency<br><br>Contact with team

		rinigng all the time about things. I think that might help everybody be a bit more reassured, even if it's just an abbreviated clinic, you don't need to see everyone just, you know. P5<br><br>It would be nice if one of the CF nurses like you said like even if it's temporary, one of them had like a mobile like a work mobile that you know we have like maybe a kaftrio hotline. I don't know if whatever you wanna call it, but it just it sometimes, It's very scary when send an email and sometimes you don't hear anything back till the next day. P4<br><br>It'd be really handy to have a number we could call just for some reassurance there and then straight away that you could, you know, you could get a call back within a couple of hours or, you know, realistically that day. And because you know not being able to contact someone quite scary. P4<br><br>Any business case they need to go to the management to say we need more here, particularly if we're all gonna be more demanding because all our little ones are going on new medications and that's scary. And we're gonna have questions and worries and need more from them for things fingers crossed for a temporary bit of time. So if they can put together, you know, to get funding for more staff. P5<br><br>What would have been nice going back and even going forward, if it's gonna be more of a delay, it's like a weekly update from the team. P4<br><br>You know here is where we are we we're expecting delivery this date we had a meeting with Vertex this day here as where we up to this week we're expecting it this date. But I bet it's the team are taking up so much time individually messaging hundreds of emails, asking the same question. P4<br><br>I just feel like too much different information from different people in the team. I feel like I get different answers. P6<br><br>Then that you're gonna have to go sooner and every month or so just for the extra blood tests. You know it's it's another added pressure on the normal daily stuff. There is pros in this cons as well. P7<br><br>I don't know what everybody else is, but to wait and see, the various members of the team and because you're going over the same point, sometimes two and three times, rather than just	Contact with team<br><br>Clinical commitment/ demand<br><br>Regular communication<br><br>Clear communication<br><br>Clear communication<br><br>Frequency of initial monitoring<br><br>Making every contact count in clinic/ timely appointments<br><br>Dietetic advice<br><br>Dietetic advice

		getting everybody in the same room and discussing it at once, because to have a young child there for 2-3 hours sometimes. P9<br><br>I would like the dietitians right to I don't know, have some sort of plan in place already. Maybe about what happens if this happens? What happens if this starts to go like this? I don't know, just some pointers as to what to look out for. What isn't ideal and when to seek help because I'm not really sure where the line is for that sort of stuff. P8<br><br>But my little boys are fussy eater. So you know it's good enough saying give him this. Give him that to this, but in actual fact, that's not gonna happen. So I think give you more of a variety of foods that you can have or you know like do put a plan together or give out leaflets with ideas on. P7<br><br>Think maybe I don't know a bit of a questionnaire to see, you know, like we do these meetings and, you know, picked between 2:00 and 3:00 to have sort of clinic times that suit their families.P7<br><br>Just to have them all come in, the one appointment at a time. So they all come in rather than come in after each other. P7<br><br>I don't know how people feel about it, but an actual home setting visit from the physiotherapist because, you know, laid in a hospital bed or when it coughs, it's totally different from coming here in your home. P8<br><br>Joey inflamed throat is the day and I wanted to take him up to Open Access at the hospital, but because it's not directly related to his CF, his breathing, they couldn't see him. But how do we know going forward what reactions they could have from kaftrio? So for this period should be able to take them if we feel they need to be seen. P9<br><br>But that's because I think the the world's will be a bit grey. How do we know if it is or it's not related? P9<br><br>I know that like overnight is probably a bit of an issue, but you know during weekends or like each day because I know that our CF Nurse doesn't , she works three days she works 3 long days but she works three days so you know I think if Cassy's ill on a Monday or Wednesday she she,	Logistic of frequent appointments/ demand on families<br><br>Making every contact count in clinic/ timely appointments<br><br>Home visits<br><br>Differentiate between symptoms<br><br>Differentiate between symptoms<br><br>Regular contact<br><br>Clear communication re availability/ access to medication<br><br>Clear communication<br><br>Clear / regular communication<br><br>Observations post giving kaftrio

		she you can't we can't can't speak to her. We can speak to another nurse, but not as CF person. So is there gonna be someone who's actually gonna be on call that we can contact? P8<br><br>I feel like I'm. I mean, it was licensed in November. And now, nearly in February, and I know that there was Christmas in the middle of that, and but it's like being drip fed. P8<br><br>We also got and very conflicting advice from and our local hospital doctor and rertiary centre doctor that we see because one of them said that once it's licence she'll be on it within a matter of days and, and, one of them said it will be it could be a couple of months. P8<br><br>I just feel like there hasn't actually been that much information about the process to get it to physically get it in your hands. P8<br><br>I think it, just a clinic or even given in clinic to then, you know, see how maybe the first hour is and then. Send them home and carry. P7<br><br>I genuinely do believe they will go above and beyond what they're paid for. What they're you know, what they have to do. We believe that they genuinely care about the well being of not only Charlie, but the rest of our family, and that comes through at every single appointment we go to P5<br><br>Yeah, they like they like I think because we lived there for so long that the start as well like a lot of them, feel like they're weird kind of family. P4<br><br>Didn't mind them having it, You know, it's it's to make sure there's no negative side effects during it P7<br><br>Yeah, I think like a lot of the side effects I've seen like where they've come from, it's being like I follow quite a few and I talked to quite a few adults online. P3<br><br>Those who've gone before us and thankful to those who took part in the clinical trials, you know before it was licensed, even, you know that have helped give us this opportunity the Grown Ups, the adults that had it before you know when it really was, they really were the Guinea pigs and just so grateful to those people that took, you know, those real risks as the first people to give it a whirl. P5	Gratitude to clinical teams<br><br>Extended family<br><br>Regular monitoring endorsement<br><br>Online forums<br><br>Thankful to other PwCF<br><br>CF community impacted – NICE statement<br><br>Not alone<br><br>Peer-support<br><br>Peer-support<br><br>Peer-support/ relatable<br><br>Support

		That day, the CF community on that Friday afternoon and for the CF trust to drop a bombshell like they normally do on a Friday afternoon. I think the CF community just went crazy like Unreal, wasn't it? P4<br><br>it’s quite reassuring knowing everybody else is worried about that, even if no one has an answer, it’s knowing that you are all in it together. P2<br><br>I'm not to look too much online, but I think as a parent, sometimes you can't help it because it's only another parent that can relate to you. P9<br><br>Somebody might be struggling with something and somebody else's, as already thought about and got away round it or, you know, just tips that you can help each other out with. And I think maybe that would count down some of the apprehension. P9<br><br>We're all in the same boat. Well, there's nobody else in that boat with you. You know nobody else will remember every single day at that hospital whether you spent, you know, six weeks in there or six month, nobody else will remember that apart from you and and somebody else's been through it. P9<br><br>And it's, it's really nice to meet two people online who have also gone through that P8<br><br>Is cause it at certain times along the way you think because you feel so much anger at times and then you feel selfish for feeling like that. But unless you're talking to somebody else, you can understand, then I don't think anybody else can fully ,they only see from the outside. P9<br><br>it's a minefield trying to juggle everything and yeah it's gonna it's gonna be annoying but totally worth it to make sure that actually everything's on track with kaftrio. But yeah, it's one of those things that only medical parents actually really understand. If they're trying to hold down a job or look after other children as well, and then do all the all the navigating a hospital appointment constantly. P8<br><br>I actually meeting up and just going to be in the same room as other CF parents. I think it's so important it's a massive thing about CF that you you're never gonna meet up with the with your kids. You you're just not so. I mean, thankfully, social media is out there for that sort of thing. P8<br><br>Just going and talking to some other people in the same boat. P8	Relate to other parents<br><br>Relate to other parents<br><br>Peer-support<br><br>Relate to others<br><br>Isolating<br><br>Isolating

		Just makes you feel like you're not alone, doesn't it? P9<br><br>it's, It's so isolating having a child with CF. It's so isolating, P9	
